# Supplementary material for: Understanding heterogeneous mechanisms of heart failure with preserved ejection fraction through cardiorenal mathematical modeling
Source: PLoS Comput Biol. 2023 Nov 13;19(11):e1011598. doi: 10.1371/journal.pcbi.1011598 (PMC10703410; doi:10.1371/journal.pcbi.1011598)
Supplement: S2 Text — (DOCX) [file pcbi.1011598.s002.docx]

**Technical Implementation**

The time scale for the cardiac cycle is on the order of seconds, while the time scale for fluid regulation by the kidney is on the order of minutes to hours to days, and the time scale for cardiac remodeling is on the order or weeks to months. To accommodate these different time scales while keeping computation times feasible, a scaling factor (SF) was introduced in the code to scale all time constants and rate constants. The model time units can be scaled from second, minutes, or hours by setting SF to 1/60, 1, or 60 respectively.

In this study, we were interested in long-term changes in cardiac filling pressures, cardiac output, chamber remodeling, etc. Changes in the cardiac cycle (stroke volume, pressures, etc) were expected to be undetectable beat-to-beat, but to accumulate over days to weeks. Thus, it is not necessary to calculate every heart beat. Instead, for all simulations in this study, we set SF = 60, which meant that 1 cardiac cycle was approximately 1 hour of simulation time, rather than 1 second. We found this to be a good compromise that allowed simulation of long time periods (e.g. over 2 years in Figure 7) with a reasonable computation time. The computation time for one year of simulation time (365x24 = 8760 time units; 7509 heart beats with heart rate = 70 bpm) on a laptop with an Intel Core i7 processor was six minutes. For multiple simulations (e.g. SOBOL), computation was distributed across multiple cores.

Note that if one were interested in understanding changes in cardiac function over shorter time periods (e.g. acute response to a vasodilator, response to exercise, etc), it would be necessary to set SF to a smaller value.
