# Supplementary material for: Understanding heterogeneous mechanisms of heart failure with preserved ejection fraction through cardiorenal mathematical modeling
Source: PLoS Comput Biol. 2023 Nov 13;19(11):e1011598. doi: 10.1371/journal.pcbi.1011598 (PMC10703410; doi:10.1371/journal.pcbi.1011598)
Supplement: S3 Text — (DOCX) [file pcbi.1011598.s003.docx]

**Pressure-Stress-Strain relationships in spherical thick-walled strain-stiffening vessels**

For a spherical, strain-stiffening (i.e. stiffness increases with increasing stretch) vessel with a given inner and outer radius, r_i_ and r_o_, the circumferential (passive) wall stress is given by:

$\sigma=P_{i}\left( \frac{r_{i}^{2}}{r_{0}^{2}-r_{i}^{2}} \right)\left( 1-\frac{r_{0}^{2}}{r_{i}^{2}} \right)$ (S72)

Thus, passive wall stress increases linearly with pressure. It also increases with inner chamber radius r_i_ and decreases as wall thickness (difference between inner and outer radii, r_i_ and r_o_). Put more simply, for a particular heart geometry, then, passive wall stress is always linearly proportional to filling pressure:

$\sigma\propto P_{i}$ (S73)

Passive stress is nonlinearly related to passive wall strain ε by the stiffness constant c_f_.

$\sigma= \beta e^{c_{f}\in}$ *(S74)*

Solving for strain and applying Eq. 18, strain is inversely proportional to stiffness, and log-linearly proportional to pressure.

$\varepsilon=\frac{\log\left( \frac{\sigma}{\beta} \right)}{c_{f}}$ *(S75)*

$\varepsilon\propto\frac{\log\left( P_{i} \right)}{c_{f}}$ *(S76)*

Thus, when filling pressure increases, wall stress will always increase proportionally (Eq. S73). For a given stiffness, strain will also increase. But if stiffness is increased as well, then strain will only increase if the log-increase in pressure is greater than the increase in stiffness (Eq. S76).
