# Supplementary material for: Understanding heterogeneous mechanisms of heart failure with preserved ejection fraction through cardiorenal mathematical modeling
Source: PLoS Comput Biol. 2023 Nov 13;19(11):e1011598. doi: 10.1371/journal.pcbi.1011598 (PMC10703410; doi:10.1371/journal.pcbi.1011598)
Supplement: S1 Table — (DOCX) [file pcbi.1011598.s004.docx]

**Table S1. Cardiac Model Parameters**

| - **Parameter** | - **Definition** | - **Value** | - **Units** | - **Source** |
| --- | --- | --- | --- | --- |
| - β | - Myocardial stiffness scaling constant | - 900 | - Pa |  |
| - $\Delta d_{max}$ | - maximum increase in myocyte diameter | - 25 | - µm | - (1-3) |
| - $\Delta l_{max}$ | - maximum increase in myocyte length | - 115 | - µm | - (1-3) |
| - $\sigma_{f,ED,0}$ | - end diastolic stress threshold for eccentric remodeling | - 4.5 | - kPa | - End diastolic stress under baseline conditions |
| - $\sigma_{f,peak,0}$ | - peak systolic stress threshold for concentric remodeling | - 49.2 | - kPa | - peak systolic stress under baseline conditions |
| - D_myo0_ | - myocyte diameter | - 23.3 | - µm | - Calculated from N_myo_, L_myo0_, V_wo_ - (1-4) |
| - HR | - heart rate | - 70 | - beats/min |  |
| - k_ven,target_ | - rate constant for venous volume link renal and cardiac submodels | - 1 | - /min |  |
| - K_d0_ | - rate constant for the increase in myocyte diameter in response to peak systolic stress | - 43.8 | - µm/year | - estimated |
| - K_l0_ | - rate constant for the increase in myocyte length in response to end diastolic stress | - 17.5 | - µm/year | - estimated |
| - L_myo0_ | - myocyte length | - 115 | - µm | - (1-4) |
| - N_myo_ | - number of myocytes | - 3.3e9 |  | - (1-3) |
| - V_f_ | - LV fibrosis volume | - 4.8 | - mL | - (1, 5) |
| - V_IS_ | - LV interstitial tissue volume | - 26.4 | - mL | - (1, 5) |
| - V_LV0_ | - unpressurized LV chamber volume | - 52 | - mL | - (1, 5) |
| - V_w0_ | - LV wall volume | - 120 | - mL | - (1, 5) |

| **Parameter** | **Definition** | **Value** | **Units** |  |
| --- | --- | --- | --- | --- |
| - *A_myo_/A_total_* | - Myocardial myocyte volume fraction | 0.76 | - |  |
| - *A_ecm_/A_total_* | - Myocardial extracellular matrix volume fraction | 0.24 | - |  |
| - β | - Myocardial stiffness scaling constant | 9 | kPa |  |
| - C | - cardiac contractility | 1 | - |  |
| - c_f_ | - LV stiffness along the fiber | 12 | - |  |
| - c_r_ | - LV radial stiffness | 9 | - |  |
| C_f,RV_ | - RV stiffness along the fiber | 9 | - |  |
| C_r,RV_ | - RV radial stiffness | 9 | - |  |
| Δd_max_ | - maximum increase in myocyte diameter | 25 | µm |  |
| Δl_max_ | - maximum increase in myocyte length | 115 | µm |  |
| D_myo,0_ | - myocyte diameter | 23.3 | µm |  |
| HR | - heart rate | 70 | beats/min |  |
| K_d_ | - rate constant for the increase in myocyte diameter in response to peak systolic stress | 43.8 | µm/year |  |
| K_l_ | - rate constant for the increase in myocyte length in response to end diastolic stress/strain | 17.5 | µm/year |  |
| *λ_f,ED,0_* | - upper limit of normal end diastolic longitudinal wall stretch | 1.16 | - |  |
| L_myo,0_ | - Baseline myocyte length | 115 | µm |  |
| l_s,a0_ | - Sarcomere length below which active stress becomes zero | 1.9 | µm |  |
| l_s,ar_ | - Sarcomere length to which the reference stress σ_ar_ is referenced to | 2 | µm |  |
| N_myo_ | - Number of myocytes | 3.3e9 | - |  |
| σ_ar_ | - Active stress scaling constant | 55 | kPa |  |
| σ_f0_ | - Longitudinal passive stress at zero stretch | 0.9 | kPa |  |
| σ_f,ED,0_ | - end diastolic stress threshold for eccentric remodeling | 4.5 | kPa |  |
| σ_f,peak,0_ | - peak systolic stress threshold for concentric remodeling | 49.2 | kPa |  |
| σ_r0_ | - Radial passive stress at zero stretch | 0.2 | kPa |  |
| - t_d_ | - Ventricle excitation fall time | 0.2 | sec |  |
| - t_r_ | - Ventricle excitation fall time | 0.2 | sec |  |
| - v_0_ | - Unloaded sarcomere shortening velocity | 50 | µm/s |  |
| - V_f_ | - LV fibrosis volume | 4.8 | mL |  |
| - V_lv,0_ | - Normal LV chamber volume under zero-pressure conditions | 52 | mL |  |
| - V_w0_ | - Baseline LV wall volume | 120 | mL |  |
| - V_w,rv_ | - RV wall volume | 100 | mL |  |

1. Olivetti G, Melissari M, Balbi T, Quaini F, Cigola E, Sonnenblick EH, et al. Myocyte cellular hypertrophy is responsible for ventricular remodelling in the hypertrophied heart of middle aged individuals in the absence of cardiac failure. Cardiovasc Res. 1994;28(8):1199-208.

2. Tracy RE, Sander GE. Histologically measured cardiomyocyte hypertrophy correlates with body height as strongly as with body mass index. Cardiol Res Pract. 2011;2011:658958.

3. Olivetti G, Cigola E, Maestri R, Corradi D, Lagrasta C, Gambert SR, et al. Aging, cardiac hypertrophy and ischemic cardiomyopathy do not affect the proportion of mononucleated and multinucleated myocytes in the human heart. J Mol Cell Cardiol. 1996;28(7):1463-77.

4. Zafeiridis A, Jeevanandam V, Houser SR, Margulies KB. Regression of cellular hypertrophy after left ventricular assist device support. Circulation. 1998;98(7):656-62.

5. Beltrami CA, Finato N, Rocco M, Feruglio GA, Puricelli C, Cigola E, et al. Structural basis of end-stage failure in ischemic cardiomyopathy in humans. Circulation. 1994;89(1):151-63.
