# Supplementary material for: Understanding heterogeneous mechanisms of heart failure with preserved ejection fraction through cardiorenal mathematical modeling
Source: PLoS Comput Biol. 2023 Nov 13;19(11):e1011598. doi: 10.1371/journal.pcbi.1011598 (PMC10703410; doi:10.1371/journal.pcbi.1011598)
Supplement: S2 Table — (DOCX) [file pcbi.1011598.s005.docx]

**Table S2. Circulatory Model Parameters**

| **Parameter** | - **Definition** | **Value** | **Units** |
| --- | --- | --- | --- |
| R_art0_ | - arterial resistance | 5e6 | Pa-s/m^3^ |
| R_per0_ | - peripheral vascular resistance | 1.27e8 | Pa-s/m^3^ |
| R_ven_ | - venous resistance | 5e6 | Pa-s/m^3^ |
| R_mitral_ | - Mitral valve resistance | 1e6 | Pa-s/m^3^ |
| R_pulm,art_ | - pulmonary arterial resistance | 3e6 | Pa-s/m^3^ |
| R_pulm,ven_ | - pulmonary venous resistance | 6.4e6 | Pa-s/m^3^ |
| R_RA_ | - right atrial resistance | 1e6 | Pa-s/m^3^ |
| V_art0_ | - arterial volume at zero transmural pressure | 450 | mL |
| V_per0_ | - peripheral volume at zero transmural pressure | 420 | mL |
| V_ven0_ | - venous volume at zero transmural pressure | 3000 | mL |
| V_pulm,art0_ | - pulmonary arterial volume at zero transmural pressure | 40 | mL |
| V_pulm,ven0_ | - pulmonary venous volume at zero transmural pressure | 250 | mL |
| V_RV0_ | - right ventricle volume at zero transmural pressure | 75 | mL |
| V_w,RV_ | - right ventricle wall volume | 100 | mL |
| C_art_ | - arterial compliance | 1.1e-8 | m^3^/Pa |
| C_per_ | - peripheral vascular compliance | 1e-8 | m^3^/Pa |
| C_ven_ | - venous compliance | 1.8e-7 | m^3^/Pa |
| C_art,pulm_ | - arterial compliance | 2e-8 | m^3^/Pa |
| C_per,ven_ | - peripheral vascular compliance | 1.65e-7 | m^3^/Pa |
| I_art_ | - arterial **inertance** | 60 | kPa-s/m^3^ |
| I_pulm_ | - pulmonary arterial **inertance** | 60 | kPa-s/m^3^ |
