# Supplementary material for: Understanding heterogeneous mechanisms of heart failure with preserved ejection fraction through cardiorenal mathematical modeling
Source: PLoS Comput Biol. 2023 Nov 13;19(11):e1011598. doi: 10.1371/journal.pcbi.1011598 (PMC10703410; doi:10.1371/journal.pcbi.1011598)
Supplement: S3 Table — (DOCX) [file pcbi.1011598.s006.docx]

**Table S3. Renal Model Parameters**

| **Parameter** | **Definition** | **Value** | **Units** |
| --- | --- | --- | --- |
| β_t_ | Tubular compliance | 0.2 | - |
| η_Na, CNT-CD_ | Fractional rate of CNT/CD Na+ reabsorption | 0.827* | - |
| η_Na, DCT_ | Fractional rate of DCT Na+ reabsorption | 0.5 | - |
| η_Na, ALH_ | Fractional rate of PT Na+ reabsorption through PT NHE3 | 0.8 | - |
| η_Na, reabs-PT,NHE3_ | Fractional rate of PT Na+ reabsorption through PT NHE3 | 0.3 | - |
| η_Na, reabs-PT,other_ | Fractional rate of PT Na+ reabsorption through non-NHE3, non-SGLT2 mechanisms | 0.35 | - |
| Φ_Na,ALH0_ | Rate of sodium delivered to the ALH under baseline conditions | 2.02* | µl/min |
| Φ_Na,intake_ | Sodium intake rate | 100 | mEq/day |
| B | LoH flow dependence coefficient | 0.75 | - |
| C_glu_ | Plasma glucose concentration | 5 | mmol/L |
| C_prot_ | Plasma protein concentration | 7 | g/dl |
| d_aa0_ | Nominal afferent arteriole diameter | 11 | µm |
| d_ea0_ | Nominal efferent arterial diameter | 16.5 | µm |
| D_c,cnt-cd_ | Connecting tubule/collecting duct effective diameter at control pressure | 22 | µm |
| D_c,dct_ | Distal convoluted tubule diameter at control pressure | 17 | µm |
| D_c,lh_ | Loop of Henle diameter at control pressure | 17 | µm |
| D_c,pt_ | Proximal tubule diameter at control pressure | 27 | µm |
| Kf | Glomerular ultrafiltration coefficient | 4 | L/min-mmHg |
| L_aa_ | Average afferent arteriole length | 73.6* | µm |
| L_ea_ | Average efferent arteriole length | 73.6* | µm |
| L_CNT-CD_ | Connecting tubule/collecting duct effective length | 10 | mm |
| L_dct_ | Distal convoluted tubule length | 5 | mm |
| L_LoH,Asc_ | Ascending loop of Henle length | 10 | mm |
| L_LoH,Desc_ | Descending loop of Henle length | 10 | mm |
| L_pt,s1_ | Length of the PT S1 segment | 5 | mm |
| L_pt,s2_ | Length of the PT S2 segment | 5 | mm |
| L_pt,s3_ | Length of the PT S3 segment | 4 | mm |
| N_nephrons_ | Number of nephrons | 2e6 | - |
| [Na]_ref_ | Normal blood/IF equilibrium sodium concentration | 140 | mmol/L |
| Na_stored_ | Maximum peripherally stored sodium | 2000 | Mmol |
| P_c,cnt-cd_ | CNT/CD control pressure | 5 | mmHg |
| P_c,dt_ | DCT control pressure | 6 | mmHg |
| P_c,lh,asc_ | Ascending loop of Henle control pressure | 7 | mmHg |
| P_c,lh,desc_ | Descending loop of Henle control pressure | 8 | mmHg |
| P_c,pt,s1_ | PT S1 segment control pressure | 20.2 | mmHg |
| P_c,pt,s2_ | PT S2 control pressure | 15 | mmHg |
| P_c,pt,s3_ | PT S3 control pressure | 11 | mmHg |
| Q_water_ | Rate constant for water transfer between blood and IF | 1 | 1/min |
| Q_Na_ | Rate constant for sodium transfer between blood and IF | 1 | 1/min |
| Q_Na,storage_ | Rate constant for sodium storage/release from the peripheral compartment | 0.1 | 1/min |
| r_glu,s12_ | Rate of glucose reabsorption through SGLT2 in the PT S1 and S2 segment per unit length | 0.2 | mmol/min/ mm |
| r_glu,s3_ | Rate of glucose reabsorption through SGLT1 in the PT S3 segment per unit length | 0.025 | mmol/min/ mm |
| R_preaff,0_ | Nominal preafferent arteriole resistance | 14 | mmHg-min/L |
| µ | Blood viscosity | 5e-7 | mmHg-min |
| Water_in_ | Water intake rate | 2.1 | L/day |

*Value calculated based on other parameters under baseline conditions
