# Supplementary material for: Understanding heterogeneous mechanisms of heart failure with preserved ejection fraction through cardiorenal mathematical modeling
Source: PLoS Comput Biol. 2023 Nov 13;19(11):e1011598. doi: 10.1371/journal.pcbi.1011598 (PMC10703410; doi:10.1371/journal.pcbi.1011598)
Supplement: S5 Table — (DOCX) [file pcbi.1011598.s008.docx]

**Table S5. Renin Angiotensin Aldosterone System model parameters**

| **Parameter** | **Definition** | **Value** | **Units** |
| --- | --- | --- | --- |
| ACE | ACE activity | 47.65* | /min |
| Chymase | Chymase activity | 2.5* | /min |
| K_AT1_ | AT1-receptor binding rate | 12.1* | /min |
| K_AT2_ | AT2-receptor binding rate | 4* | /min |
| K_d,AngI_ | AngI degradation rate | 0.0924 | /min |
| K_d,AngI_ | AngII degradation rate | 0.146 | /min |
| K_d,AT1_ | AT1-bound AngII degradation rate | 3.47 | /min |
| K_d,renin_ | Renin degradation rate | 4 | /min |
