# Supplementary material for: Understanding heterogeneous mechanisms of heart failure with preserved ejection fraction through cardiorenal mathematical modeling
Source: PLoS Comput Biol. 2023 Nov 13;19(11):e1011598. doi: 10.1371/journal.pcbi.1011598 (PMC10703410; doi:10.1371/journal.pcbi.1011598)
Supplement: S6 Table — (DOCX) [file pcbi.1011598.s009.docx]

**Table S6. Model Initial Conditions**

| **Variable** | **Definition** | **Value** | **Units** |
| --- | --- | --- | --- |
| AngI | Angiotensin I | 8.164 | pg/mg |
| AngII | Angiotensin II | 5.17 | pg/mg |
| AT1-bound AngII | AT1-bound AngII | 16.6 | pg/mg |
| AT2-bound AngII | AT2-bound AngII | 5.5 | pg/mg |
| CO | Cardiac Output | 5 | L/min |
| IFV | Interstitial Fluid Volume | 12 | L |
| Na_blood_ | Blood sodium amount | 700 | mEq |
| Na_IF_ | Interstitial sodium amount | 2100 | mEq |
| Na_stored_ | Stored sodium amount | 0 | mEq |
| PRC | Plasma Renin Concentration | 17.84 | pg/ml |
| V_art_ | Arterial volume | 573 | mL |
| V_b_ | Blood Volume | 5 | L |
| V_LV_ | LV chamber volume | 110 | mL |
| V_RV_ | RV chamber volume | 182 | mL |
| V_per_ | Peripheral circulation volume | 441 | mL |
| V_pulm,art_ | Pulmonary arterial volume | 123 | mL |
| V_pulm,ven_ | Pulmonary venous volume | 476 | mL |
| V_venous_ | Venous volume | 3278 | mL |
