# Supplementary material for: Understanding heterogeneous mechanisms of heart failure with preserved ejection fraction through cardiorenal mathematical modeling
Source: PLoS Comput Biol. 2023 Nov 13;19(11):e1011598. doi: 10.1371/journal.pcbi.1011598 (PMC10703410; doi:10.1371/journal.pcbi.1011598)
Supplement: Attachment — Submitted filename: response to reviewers_v2.docx [file pcbi.1011598.s010.docx]

We thank the reviewers for their time and energy in reviewing this paper. We believe the comments and suggestions are thoughtful, fair, and add greatly to the quality of the manuscript. We have attempted to address each of them to the best of our ability.

**Reviewer #1**

**In this manuscript, the authors describe a series of simulations made in order to examine factors that produce heart failure with preserved ejection fraction (HFpEF). The authors sought to leverage this integrative model to understand the diverse manifestations of HFpEF, and in particular its relationship with the other main heart-failure modality, HFrEF. They ultimately propose a unifying hypothesis of a strain-based remodeling signal as being the key mediator of the HFpEF vs. HFrEF paradigm.

This manuscript is well-written and carefully reasoned. It offers an interesting a meaningful perspective by employing an integrative cardiovascular model in a series of strategically constructed simulations. I have only a few comments, which I believe are important, but which should be straightforward to address.

1. The mathematical definition of HFpEF did not entirely make sense. Certainly, elevated LVEDP and EF >50% are necessary and logical, but would it not also be important to manifest a drop in cardiac output? This is, after all, why heart failure matters. In Figure 5, CO is examined but perhaps should be given greater importance in the overall picture? This matter is somewhat more critical in the context of the later simulations on remodeling. In the parameter regime used to illustrate maintained HFpEF in spite of the potential for LV remodeling, does this yield a meaningful reduction of CO (such that this virtual patient would have noticeable heart failure)?**

Response: Thank you for this comment. In this study, we aimed to simulate resting chronic heart failure. The reason that we did not include a reduction in CO in the definition of HF-pEF is that, perhaps somewhat counterintuitively, resting CO is often actually quite normal in chronic HF-pEF patients. For instance, in the PROMIS-HFpEF study ((1) Table 2) of HF-pEF patients with and without coronary microvascular dysfunction (CMD), mean CO was normal (5.1 L/min) in HF-pEF patients without CMD, and only slightly reduced (4.7 L/min) in the group with CMD. This is pretty typical of studies of HFpEF, and studies of HFrEF have also shown that most chronic HFrEF patients also have normal resting CO.

The problem in heart failure is that the normal CO can only be maintained at the expense of increased filling pressures (LVEDP), and this is the key feature we aimed to model. Essentially, when something happens that causes CO to tend to decrease, blood pressure and pressure into the kidney also decreases, which causes the kidneys to retain sodium and water, increasing blood volume, which in turn increases cardiac filling pressures (LV EDP) (see also the response to Comment #2). The increase in cardiac preload then increases stroke volume (SV) through the frank starling mechanism, thus normalizing CO. So resting CO is maintained but only at the expense of higher cardiac filling pressure.

Most of the signs and symptoms of heart failure are the consequence of elevated filling pressures (edema, shortness of breath, the feeling of “drowning” that ultimately sends patients to the ER and results in hospitalization). Thus, we did not include CO in the criteria for HFpEF because we do not expect resting CO to be reduced in most HFpEF patients.

One thing we did not consider in this definition of HF-pEF is impaired exercise capacity– even though CO is normal at rest, HF patients often can’t increase CO to meet increased demands when they exercise. HFpEF patients with impaired exercise capacity but normal filling pressure have much better (near age-normal) prognoses compared to patients with elevated filling pressures (2) . Therefore, we believe it is reasonable to focus on increased filling pressures as the key factor in determining HFpEF patients with increased risk for morbidity/mortality. As we state in the discussion, in future work we do plan to investigate factors that influence cardiac reserve and exercise capacity in the future.

We have added an explanation of the lack of consideration of CO in the methods section, under “Mathematical Definition of HFpEF”

**2. As a specific way of addressing the above comment, would it be possible to add CO to Figure 7, just to show that there is significant reduction in CO for both HFpEF and HFrEF outcomes?**

Response: The new figure is also shown below. As the top right panel shows, resting CO remains normal or near normal for a long time in both HFpEF and HFrEF, but LV EDP and interstitial fluid volume increases progressively. As described in response to the previous comment, normal resting CO is maintained through an increase in cardiac filling pressure through fluid retention.

**Minor Comments:
1. I believe that in the introduction, line 14, the word ‘population’ is extraneous.**

Corrected.

**2. Under ‘Software Implementation’, I believe that the article (“a”) preceding R v4.1.1 is unnecessary.**

Corrected

**Reviewer #2**

**The manuscript, PCOMPBIOL-D-23-00308, “Understanding heterogeneous mechanisms of heart failure with preserved ejection fraction through integrative cardiorenal mathematical modeling” by Basu et al., seeks to use computational modeling to determine whether one or many insults lead to heart failure with preserved ejection fraction (HFpEF). The authors find that passive stiffness is an important modulator but a ‘second hit’ is required to obtain elevated LV filling pressures (in contrast to hypertrophic patients). More than one mechanism can act as this hit.

Overall, the model provides a result that can be of substantial interest to both pre-clinical and clinical researchers working to understand HFpEF.

MAJOR COMMENTS:

1. Discussion. The discussion is overall long and difficult to follow.**
**1a. The discussion (and possibly the introduction) may be reduced or revised. I.e. there appear to be repetitive statements about the HFpEF phenotype. Potentially repetitive statements about the results (multiple references to what was or was not related to increased LVEDP.**

Response: We agree there was some duplication in the discussion of points made earlier. We have tried to condense the discussion and remove parts that are repetitive. However, we have also added some points to the discussion based on other reviewer comments, so while parts were condensed, it may not appear shorter.

**1b. Could the new equations (Eq.17-21) instead be included in the supplemental materials?**

Response: We agree and we condensed this section down and moved most of the equations to the supplement. We think that the two remaining equations are necessary to make the point of this paragraph and have left them in the main text. But if the reviewer believes these should be moved as well, we are open to moving them as well.

**1c. The most pressing comment from this reviewer is that many of the comments on the heterogeneity of HFpEF are written with a different language than the multiple components of the model- as if the model aspects and physiology were written separately. i.e. this reviewer was unable to easily identify that, because multiple interventions WITH increased LV stiffness produced increased LVEDP, a heterogeneous phenotype COULD lead to HFpEF symptoms as defined by the authors.**

Response: I am not sure we fully which section the reviewer is referring to, but I think this may be referring to the section of the discussion where we attempted to link the various co-morbidities with HFpEF with its heterogeneity. In the original draft we did not do a good job of first linking the general comorbidities to the specific pathophysiological mechanisms we tested, and this probably made it difficult to follow the point we were trying to make. We have edited this section of the discussion and attempted to clarify this.

The updated text reads:

- “HF-pEF is associated with multiple co-morbidities (kidney disease, metabolic syndrome, coronary artery disease, atrial fibrillation, valvular disease) (3), and each of these comorbidities may contribute insults such as the ones evaluated here (hypertension, impaired contractility, vascular stiffening, etc.). Our simulations suggest that comorbidities that produce cardiac or vascular changes that are not severe enough to cause HFrEF in a heart of normal stiffness may cause HF-pEF as LV stiffness is increased. Understanding HFpEF mechanisms in this way, as additional insults added to an already stiffened myocardium, provides an important step forward in the path to understanding and reproducing the heterogeneity within the HFpEF population.”

**2. Methods- Eq.8. There are two concerns related to this formulation:
2a. This formulation is different than what is found in Bovendered (ref 47). If it is adapted in prior work from the authors, please reference it here.**

We thank the reviewer for this comment, and have tried to make this more clear in the text. The formulation used here is an extension of the equation from Bovendeerd et al. In Bovendeerd et al the excitation signal is always symmetric. Here, we have updated the equation to allow the falling side of the signal to be longer than the rising side, in order to simulate slower cardiac relaxation. However, the formulation given here can be shown to collapse down to the same equation in Bovendeerd (ref 47, eq. 12 ) when Δt_f_ = 0 and t_r_ = t_d_ = 0.5t_twitch_(symmetric rise and fall).

From Bovendeerd (eq. 12):

$$g\left( t \right)= \left\{ \begin{matrix} {sin}^{2} \left( \pi\frac{t}{t_{twitch}} \right), \\ 0 \end{matrix} \right.\begin{matrix} t<{t_{\mathrm{twitch}}} \\ t_{twitch}\leq t \leq t_{beat} \end{matrix}$$

Manuscript, eq. 8:

$g\left( t \right)= \left\{ \begin{matrix} {sin}^{2} \left( \pi\frac{t}{t_{twitch}} \right), & t<t_{r} \\ {cos}^{2} \left( \pi\frac{\left( t-t_{r} \right)}{t_{twitch}+\Delta t_{f}} \right) & t_{r}\leq t\leq t_{twitch}+\frac{\Delta t_{f}}{2} \\ 0 & t_{twitch}+\frac{\Delta t_{f}}{2}\leq t \leq t_{beat} \end{matrix} \right.$

If Δt_f_ = 0 and t_r_ = t_d_ = 0.5t_twitch_ *, then the* second term is:

$${cos}^{2} \left( \pi\frac{\left( t-t_{r} \right)}{t_{twitch}} \right)=\cos^{2} \left( \pi\frac{t}{\mathrm{twitch}}-\pi\frac{t_{r}}{t_{twitch}} \right)= \cos^{2} \left( \pi\frac{t}{\mathrm{twitch}}-\frac{\pi}{2} \right)= \sin^{2} \left( \pi\frac{t}{\mathrm{twitch}} \right)$$

We have updated the text to make it more clear how this equation was changed from Bovendeerd et al and why.

**2b. The formulation also does not include contraction-relaxation coupling effects. The lack of contraction-relaxation coupling may cause a substantial difference from real-physiologic changes, especially when small or moderate changes in systolic function are trialed.**

The reviewer is correct - a limitation of this model is that it does not attempt to represent the molecular/cellular mechanisms that govern contraction and relaxation, and thus contraction-relaxation coupling is not enforced. The activation signal is prescribed by equation 8, and the strength of contraction is prescribed by the contractility parameter c in Eq. 3. For the purposes of this analysis, this allows us to impose a separate relaxation time and to test the hemodynamic consequences of slowed relaxation independently from alterations in other factors such as strength of contractility. But as the reviewer correctly points out, these mechanisms are likely not independent in reality. Because even large changes in relaxation time alone had little effect on LV EDP and EF, we don’t expect that any coupling effects not captured in the model (e.g. changes in relaxation when contractility is varied) would impact the conclusions regarding the major factors that increase LV EDP at normal EF, which is the main point of this paper. However, these mechanisms could certainly be important in other situations and applications of this model. We have added the following text to the discussion:

We have added this limitation to the discussion section.

**3a. Methods-Eq.9. Relaxation is defined using a time, not a relaxation rate (tau) as typically defined clinically and in research literature. This difference should be noted and/or the authors should refer to this specifically as IVRT, not as relaxation.**

We have added more explanation around the definition of parameters defining slowed relaxation. We have also added the calculated values for relaxation time constant tau that correspond to the range of values used for Δtf.

Reviewer #3 had a similar question, regarding the definition and units of Δtf vs the use of IVRT. In brief, IVRT (isovolumetric relaxation time) is a different measurement that Δtf . is a calculated value from the model output, and it changes as a consequence of changing Δtf, but it is not a direct function of Δtf. We calculated IVRT at different values of Δtf, in the same way that it is calculated clinically – as the time between when the aortic valve closes and the time at which the mitral valve opens – this is the time during which the pressure in the ventricle is falling while the volume is remaining constant. However, this is different from the time at which the ventricle muscle is relaxing – this begins before the aortic valve closes and continues after the mitral valve opens. But actual ventrical relaxation is difficult to measure clinically, so IVRT is often measured instead as an indirect reflection of muscle relaxation time. We provided the IVRT values to allow comparison with values typically observed in normal and HFpEF patients, in order to 1)show that changing Δtf does increase IVRT and 2) to give a sense of how the magnitude of changes in Δtf, which is not clinically measurable, relate to observed changes in IVRT, which is measureable.

The same explanation applies for tau – it is a calculated value from the model output, but when the parameter Δtf is changed, this change is reflected in changes in the calculated value for tau.

**3b. It is also concerning that the authors used a maximal tr up to 432 ms greater than 3 times the normal reported values (85-160 ms). It is unclear to this reviewer how this impacts the sensitivity analysis- could the choices of delta_tf (0.0, 0.5,1.3) impact the sensitivity analysis?**

We actually first conducted the sensitivity with a smaller range for Δtf (and thus smaller corresponding IVRTs), but we found that LV EDP was insensitive to small changes in Δtf. We decided to evaluate a wider range to see if perhaps larger changes had some effect. But as shown in the manuscript, even at extremely high, likely physiologically unlikely levels, there was very minimal effect on LV EDP.

4. **Discussion. A major limitation is the lack of validation to a physiologic dataset. While the authors do rightly reference prior validation of the models, comparison of these model results to clinical or pre-clinical research data (preferably more recent than the Grossman ref), would be ideal. A comment about this in the text is recommended.**

We thank the reviewer for this comment. We we agree that validation will be critical before this model can be used to make predictions in HFpEF. The clinical data available for validation in HFpEF is more limited than in other populations such as HFrEF, LV hypetrophy, etc. that we have modeled previously. But there are some studies which provide useful information for validation, as we describe below. We have actually conducted some of these validation analyses already. However, given the current length of this manuscript, we have chosen to put those validation studies in a follow-up manuscript that we are currently working on. The aim of this follow-up manuscript is to demonstrate that the definition and characterization of HFpEF developed in this paper can be used to create virtual populations that, when matched to baseline charactistics of clinical trials, can reproduce responses to interventions consistent with these clinical trials. Describing these validation studies is not a trivial thing, and we feel that it would just make the current manuscript too long.

We have added a statement on the current lack of validation in the limitations section.

When we have used the cardiorenal model to simulate other populations (HF-rEF, LV Hypertrophy, CKD), we have done this by generating populations of virtual patients, matching those virtual patients to the baseline characteristics of clinical trials, and then demonstrating that the virtual patients respond as expected to perturbations – usually therapeutic interventions. For HF-rEF, we reproduced the SOLVD study with the ace inhibitor enalapril. For LV hypertrophy, we reproduced the LIFE study with the angiotensin receptor blocker losartan, and for chronic kidney disease, we have reproduced multiple studies (RENAAL, IDNT, AVOID, DAPA-SALT, DAPA-CKD, etc), with ACEi, ARBs, and SGLT2 inhibitiors.

For HF-pEF, we are now doing something similar, although unfortunately there are few HFpEF studies that have reported biomarkers that are straightforward to reproduce (few mechanistic studies – mostly outcomes trials). However, there is a key study by Barry Borlaug and colleagues in which they recruited patients with either HF-pEF or HF-rEF, treated them acutely with nitroprusside while catheterized, and reported changes in variables such as CO, stroke volume, LV EDP, systolic and diastolic blood pressure, etc. This study interestingly reported similar acute drops in LV EDP in both types of patients, but much smaller increases in CO and SV in HF-pEF compared to HF-rEF. Our model has been able to reproduce this difference (

Secondly, while we cannot model clinical outcomes data (morbidity and mortality – M&M) directly, we can test our model by seeing whether its predicted changes in variables such as LV EDP are consistent with differences in outcomes. Clinical studies have consistently shown little risk reduction with ACE inhibitors, but recent trials found that SGLT2 inhibitors DO reduce M&M. We simulated ACEi and SGLT2i treatment in HFpEF and HFrEF virtual patients over 1 year, and the model predicted substantially more reduction in LV EDP and in interstitial fluid volume with SGLT2i than with ACEi. While this can not be directly compared with data, as no studies to date have reported changes in LV EDP or its surrogates over time in HFpEF, these results are quite consistent with the clinical findings.

These validation studies have recently been summarized in first author S.B.’s PhD thesis, and we are happy to share this with the reviewer upon request. We hope that this, as well as including a limitation statement on the lack of data for validation, will be considered sufficient assurance.

**5. Discussion. Another limitation is that the model does not appear to be able to remodel (i.e. Eq.2 is based on a single geometry). A comment about how remodeling (both in shape and/or mesostructured (cell/sarcomere alignment)) might impact the reported result is recommended.**

Equation 2 describes the relationship between ventricle blood volume (Vlv) and stretch λ_f_ for a given ventricle geometry defined by a particular wall volume V_w_ and zero-stretch chamber volume V_lv,zero_.

- $\lambda_{f}=\left( \frac{V_{lv}+\frac{1}{3}V_{w}}{V_{lv,zero-p}+\frac{1}{3}V_{w}} \right)^{\frac{1}{3}}$	( 2 )

However, the wall volume and chamber volume can both change over time in response to mechanical signals, as illustrated in Figure 1B and described later on page 18 under the section “Simulating HFpEF over time: evaluating mechanical drivers of cardiac remodeling”. This section summarizes the understanding of processes driving remodeling, our previous work modeling changes in sarcomere length and diameter, and then updates made to the remodeling laws in this paper. Indeed, a key finding from this analysis is that a chronic state of HFpEF can only be produced if wall STRAIN, not stress, is the mechanical signal for myocyte elongation and outward remodeling of the ventricle. If wall stress is the signal, then a state of HFpEF can only be produced temporarily, but will always progress quickly to HFrEF (Figure 7).

MINOR COMMENTS:

**6. Introduction-paragraph 3 (near refs 11-21). While the introduction includes a general summary of results of clinical trials, the authors should include a statement the HFA and ESC recommendations for HFpEF diagnosis (PMID: 31504452).**

We appreciate this suggestion, and had added a brief summary of the diagnostic guidelines in the intro.

**7. Results-Figure 8. The authors include increased stiffness with normal systolic function. It is likely that if collagen stiffness is increased, contractility will be reduced. Doe the authors have mathematical evidence of this? Should this be addressed in the text?**

In the analysis in Figure, we aimed to determined the effects when collagen stiffness, myocyte stiffness, and contractility were varied independently of each other. However, it is possible that changes in collagen stiffness may in turn elicit intracellular changes that alter myocyte stiffness or contractility. Similarly, changes in myocyte stiffness may have effects on myocyte contractility as well. Here, though, the aim was to determine their independent effects (or interdependent effects when varied together).

We have added a statement in the text to clarify that while these are varied independently here, they may not be independent in reality.

**8. This reviewer would appreciate consistent or clear formatting (indent or spacing) between paragraphs.**

We have attempted to make the spacing and indentation consistent in the final clean (non-redlined) version.

**Reviewer #3**

**The authors have used a computational model that couples cardiovascular and renal function to investigate various mechanisms associated with HFpEF. The following comments are submitted for the authors’ consideration.

1) There are several places in the manuscript, including the introduction and discussion, where a sentence was left incomplete. For example: “It also increases with inner chamber radius ri and decreases as wall thickness (difference between inner and outer radii, ri and ro).” and “…valvular disease predisposes one to development of HF, and different combinations of comorbidities are likely responsible for (9).”**

Response: Thank you for noticing these. We have corrected them, and read through the manuscript carefully and tried to find and correct any similar errors.

**2) The description of the parameter Δtf used in equation 9 is unclear in the text and seems to be inconsistent with how it is described in table 1. In the text, it says: “To evaluate the effect of slowed relaxation, the parameter Δtf was increased from 0 to 1.3, which increased IVRT from its baseline value of 80 ms up to 432 ms.” This makes it seem unitless and also would not increase the value of t_twitch to the range described. Table 1 says that Δtf has units of seconds and was used to increase t_twitch to +0-325% of its normal value. If this is the case, then even the percent ranges given in the table are inconsistent with the values of 80 ms up to 432 ms. This should be clarified and made consistent between the text and table.**

Response: We appreciate this comment and have tried to make the terminology more clear. Δtf is not unitless – it has units of seconds. This was not clear in the original manuscript. The normal value for t_twitch_ is 0.4 s. Thus, when Δtf is 1.3s, t_twitch_ is 100x1.3/.4 or 325% of normal t_twitch_. This is where the numbers in the table came from. We have tried to make this more clear in the updated text.

- IVRT (isovolumetric relaxation time) is a different measurement that Δtf . It is a calculated value from the model output, and it changes as a consequence of changing Δtf, but it is not a direct function of Δtf. We calculated IVRT at different values of Δtf, in the same way that it is calculated clinically – as the time between when the aortic valve closes and the time at which the mitral valve opens – this is the time during which the pressure in the ventricle is falling while the volume is remaining constant. However, this is different from the time at which the ventricle muscle is relaxing – this begins before the aortic valve closes and continues after the mitral valve opens. But actual ventricle relaxation is difficult to measure clinically, so IVRT is often measured instead as an indirect reflection of muscle relaxation time. We provided the IVRT values to allow comparison with values typically observed in normal and HFpEF patients, in order to 1)show that changing Δtf does increase IVRT and 2) to give a sense of how the magnitude of changes in Δtf, which is not clinically measurable, relate to observed changes in IVRT, which is measurable.
- Per another reviewers comments, we also added values for the corresponding relaxation time constant tau (another common clinical measure of relaxation that can be calculated from the model output) for the range of Δtf considered.

**3) In the text it states that the model was simulated for 60 days. Is this how long that simulation took to run, or does this mean you simulated 60 days of function, which would be 5.2 million heart beats. This needs to be clarified, i.e., how many heart beats were simulated and how long did it take. In addition, what resources were used to run the simulations, desktop with 8 cores, cluster with 128 cores, etc.**

We agree that this needs more explanation. We have updated the text to read:

“the model was simulated for 60 days (about 1 minute computation time – see supplement for more info) – a duration sufficient to allow the model to settle to a new stable state.”

And we added the following to the supplement:

Technical Implementation:

The time scale for the cardiac cycle is on the order of seconds, while the time scale for fluid regulation by the kidney is on the order of minutes to hours to days, and the time scale for cardiac remodeling is on the order or weeks to months. To accommodate these different time scales while keeping computation times feasible, a scaling factor (SF) was introduced in the code to scale all time constants and rate constants. The model time units can be scaled from second, minutes, or hours by setting SF to 1/60, 1, or 60 respectively.

In this study, we were interested in long-term changes in cardiac filling pressures, cardiac output, chamber remodeling, etc. Changes in the cardiac cycle (stroke volume, pressures, etc) were expected to be undetectable beat-to-beat, but to accumulate over days to weeks. Thus, it is not necessary to calculate every heart beat. Instead, for all simulations in this study, we set SF = 60, which meant that 1 cardiac cycle was approximately 1 hour of simulation time, rather than 1 second. We found this to be a good compromise that allowed simulation of long time periods (e.g. over 2 years in Figure 7) with a reasonable computation time. The computation time for one year of simulation time (365x24 = 8760 time units; 7509 heart beats with heart rate = 70 bpm) on a laptop with an Intel Core i7 processor was six minutes. For multiple simulations (e.g. SOBOL), computation was distributed across multiple cores.

Note that if one were interested in understanding changes in cardiac function over shorter time periods (e.g. acute response to a vasodilator, response to exercise, etc), it would be necessary to set SF to a smaller value.

**4) In the description of the stress driven remodeling law (model 1), why was growth assumed to be permanent and not reversible?**
We have reworded the text to say that “ Increases in myocyte length were approximated as irreversible, since previous work has suggested that regression of myocyte elongation may occur much more slowly than progression.

While it is clear that myocytes can increase and decrease their diameter over relatively short time periods, increases in myocyte length occur more slowly, and the clinical literature is unclear on whether myocytes can shrink back to normal length after elongating. “Reverse remodeling” certainly occurs, but it is usually defined clinically as an increase in EF or a decrease in LV EDV, and changes in both of these may be simply a consequence of reducing filling pressures and/or reducing myocyte diameter. Most treatments acutely reduce these things and are said to “reverse remodeling”– but it is unclear if it is truly reversed dilatation. Treatments generally more successful in slowing progression than reversing it.

In previous publications we have described modeling of regression of LV remodeling with ARB or ACEi therapy in two clinical studies (LV hypertrophy (LIFE study - (4) and HFrEF (5)). In both of these studies, changes in LV mass and LV volume occurred over time in placebo group, and were partially reversed and slowed in the treatment groups (Enalapril in SOLVD and Losartan in LIFE). The model could be best reproduced these findings when we assumed: 1) that increases/decreases in myocyte diameter occurred at same rate, 2) that changes in myocyte diameter occur much faster than increase in myocyte length, and 3) increases in myocyte length occurs orders of magnitude faster than any decrease. In other words, decreases in myocyte length occurred more slowly than detectable in these studies (give lengths here). Therefore, we approximate myocyte elongation as irreversible.

**5) In the simulation procedure section, it says the simulation was run for 1 year. The comment here is similar to the above. Does this mean you simulated all of the heart beats in a year? Did you do any separation of time scales, which is quite common with growth laws. Also, were the rate constants tuned to experimental data to get the time course of growth over a year?**

- For the first part of this comment, please see response above to #3 above.
- For the question about rate constant tuning, we used the rate constants which we previously calibrated to fit clinical data on cardiac growth and remodeling in patients with LV hypertrophy and HFrEF patients, described in detail in (4) and (5, 6)).
- Both of these studies used LV end diastolic stress as the driver of outward remodeling (Eq. 11). In replacing stress with strain as the driver of remodeling (eq. 12), the rate constant Kl was scaled based on the ratio of LV ED stress to strain under normal conditions. We found that this new time constant was still table to reproduce the rates of outward dilatation observed in HFrEF patients (i.e. we could still reproduce the results from Yu, Basu et al. 2020. )

**6) How were the parameter values inequations 15 and 16 selected? Also, for the normal case, it's more precise to say cf represents the combined effects of the myocytes and ecm, not that the stiffness of the myocyte and collagen are the same. They are very different.**

Β and Cf were previously determined by Bovendeerd et al for a homogeneous ventricle. We have updated the text to include this reference.

We have also updated the text to say “Under homogeneous conditions, c_f,myo_ and c_f,ecm_ were assumed to be the same” rather than “Under normal conditions c_f,myo_ and c_f,ecm_ were assumed to be the same”, since, as the reviewer points out, myocyte and collagen stiffness are very different, and thus the homogeneous case is not a “normal” case.

**7) For the sensitivity study, how many points were used in the parameter space? Were they evenly distributed or random?**

For the sobol sensitivity analysis, we used an initial sample size of N = 2^9, or 512. Since 8 parameters were varied (p = 8), the total number of parameter sets simulated was 2N*(p+1) = 9180.

**8) I am a big fan of Sobol sensitivity indices. But one must take care in using them. In figure 3, it can be seen that there are first order sensitivity indices that are larger than the total. This is not a realistic result. If you have 3 parameters, then Stotal = S1+S12+S13+S123, i.e., it is the sum of the first and higher order indices. However, it has been shown that when doing numerical approximations of the indices, with something like a monte carlo approach, you can get artifact if you do not sample enough times. Thus, comment 7 is very important in this context. How many points did you sample in the parameter space. If you did not use enough, then your indices could be errant. The authors need to explain in detail how the study was conducted and possibly consider simulating more points in the parameter space. If simulations are too long, you could consider building a segregate model.**

Thank you very much for this insightful comment. In investigating the cause for the lower Ti vs Si, we learned that this is a known problem of the Saltelli method for monte-carlo estimation of sobol indices (see (7), section 2.2). It is possible that using a much larger sample size could resolve this. However, Azzini et al proposed a newer estimator for which Ti is always > Si. This estimator involves the use of an additional input parameter matrix, but can obtain more accurate indices with a smaller sample size than other methods ((8). This method is also available in the sensobol package in R. In our updated figure, we used the Azzini method. The values calculated with Azzini et al method were numerically different from those calculated by Saltelli et al. However, the trends remained the same. In addition, the confidence intervals calculated with Azzini were smaller than with the previous method (we believe this is because the number of replicates is larger with Azzini, 2N*(P+1) = 9180, vs N*(P+2) = 5120 with Saltelli). Because the confidence intervals were smaller, some of the minor effects that were not previously significant were found to be significant (although the strength of the effect was minimally changed). We have updated the text to reflect these differences.

We have also added the following to the methods section to provide more information on how the Sobel analysis was conducted:

“The sobol analysis was conducted using the sensobol package in R (9). An initial sampling of N=29 was used, and the total number of parameter sets tested was 2N*(1+p) or 9216 for p=8 parameters. Sobol indices were estimated using the Monte-Carlo estimator of Azzini et al (8), and confidence intervals were determined by bootstrapping with 1000 replicates.”

**9) With Sobol indices, a lot of times the sampling variability in the sensitivity indices is assessed with confidence intervals. This is done by resampling the parameter space multiple times and calculating the new values for the sensitivity indices. However, in figure 3 standard deviation bars are presented. How did you assess the standard deviation? How many times were the parameters resampled?**

Thank you for noticing this. The error bars were labeled incorrectly in the original submittion – these bars are confidence intervals, not standard deviations. They were calculated by bootstrapping using 1000 replicates. We tested this using a larger number of replicates and it did not alter the results.

We have updated the text and also added information on how the confidence intervals were determined in the methods section (see above).

**10) Be careful using the word significant if you have not conducted a statistical analysis.**

We have revised the text in several places to be use this word only when referring to true statistical significance.

**11) For the results presented in figure 8, it is unclear exactly how the different stiffness parameters were altered. Were the beta values increased for the myocytes and then the ecm? One would assume so, since the cf parameter is in the exponential and would drastically alter stiffness. Additionally, it is unclear what the parameter values are for the myocytes and ecm. They should not be the same, since these constituents behave very differently.**

In this analysis, only the Cf parameter values were changed. As discussed above in response to #6, we started from the condition of homogeneity, in which both ECM and myocytes have the same stiffness relationship (same beta and Cf). Beta defines the residual stress at zero stretch, and we assume that this is unchanged. Cf defines the steepness of the stress-stretch relationship, and this is what was varied (increased by 75%), from its starting value of 11 to a new value of 19.25.

Previous unpublished analysis in our lab of data reported in a study by Chaturvedi 2010 supports these values. In this study, strips of cardiac muscle were obtained from human heart biopsies, and force-extension curves were produced for myocytes and for strips with myofilaments removed (so mostly ECM). Strips were obtained from normal, pressure-overloaded, and volume-overloaded hearts). We digitized this data and fit Eqs 16/17 to obtain Cf for each strip, and calculated the mean Cf for each condition. T

***Force-extension curves, and estimated stiffness Cf, for myocytes obtained from normal donor, pressure-overload, and volume-overload hearts.***

***Force-extension curves, and estimated stiffness Cf, for ECM strips (tissue strips with myofilaments removed) obtained from normal donor, pressure-overload, and volume-overload hearts.***

***Mean estimated stiffness constants***

In all cases except the pressure-overloaded myocytes, the estimated stiffness parameter Cf was near the value (11) previously determined by Bovendeerd et al, and used as the normal value in our study. On the other hand, the myocyte stiffness in pressure-overloaded hearts was much higher – up to 30. In our model, increasing myocyte stiffness above about 20 resulted in LV EDP values that were unphysiologically high (above 40 mmHg), so we did not increase Cf,ecm all the way to 30, but capped it at 20.

As the reviewer points out, because Cf is in the exponent, a change in Cf causes a large change in the stress-relationship. But these large differences are actually seen in the experimental data above – the slopes of the myocyte stress-stretch relationship are much much steeper in PO than in VO or normal tissue.

**Since the stress-stretch relations have the exact same functional form, if you did use the same values for both, then perturbing each one would lead to the same outcome and not distinguish the influence of each correctly. This should be stated clearly.**

While they have the same functional form, the two components have very different volume fractions. If the volume fractions were 50/50, then changing perturbing either would give the same result. But because the ECM volume fraction is much smaller than ECM, the effects of changing ECM stiffness on LV EDP, stress, and strain are different than the effects of changing myocyte stiffness. This is why we looked at each of these three components (myocyte stiffness, ecm stiffness, and ecm volume fraction) in Figure 8.

**12) No limitations are given in the manuscript. These should be noted in a subsection in the discussion.**

We have added an expliit section on limitations, including limitations not previously mentioned, as well as other parts of the discussion that addressed limitations but did not explicitly label them as such.

**Have the authors made all data and (if applicable) computational code underlying the findings in their manuscript fully available?**
The [PLOS Data policy](https://journals.plos.org/ploscompbiol/s/materials-and-software-sharing) requires authors to make all data and code underlying the findings described in their manuscript fully available without restriction, with rare exception (please refer to the Data Availability Statement in the manuscript PDF file). The data and code should be provided as part of the manuscript or its supporting information, or deposited to a public repository. For example, in addition to summary statistics, the data points behind means, medians and variance measures should be available. If there are restrictions on publicly sharing data or code —e.g. participant privacy or use of data from a third party—those must be specified.

Reviewer #1: Yes

Reviewer #2: Yes

Reviewer #3: **No:** The manuscript and supplement provide extensive details about the equations that were employed, but the code itself is not available.

Response: All code has been made available in a zip file with the submission
